# Performance of Computed Tomography Angiography Before Revascularization Is Associated With Higher Amputation-Free Survival in Rutherford IIb Acute Lower Limb Ischaemia

**DOI:** 10.3389/fsurg.2021.744721

**Published:** 2021-10-25

**Authors:** Ebba Saphir, Robert Svensson-Björk, Stefan Acosta

**Affiliations:** ^1^Department of Clinical Sciences, Lund University, Lund, Sweden; ^2^Department of Cardiothoracic and Vascular Surgery, Vascular Center, Skåne University Hospital, Malmö, Sweden

**Keywords:** acute lower limb ischaemia, motor deficit, Rutherford classification, revascularization, computed tomography angiography

## Abstract

**Background:** Acute lower limb ischemia with a motor deficit (Rutherford IIb) needs urgent revascularization to avoid major amputation and mortality. It is unclear whether immediate revascularization without performing CT angiography (CTA) prior to revascularization in Rutherford IIb acute lower limb ischemia (ALI) is associated with better outcomes.

**Methods:** Retrospective observational study of Rutherford IIb ALI patients treated between 2006 and 2018. A propensity score adjusted analysis was performed to compare outcomes after the performance of CTA examination or not.

**Results:** Among 681 patients, 260 had Rutherford IIb ALI. CTA prior to revascularization was performed in 131 (50.4%) and increased (*p* < 0.001) throughout the study period. Open vascular and endovascular surgery was first performed in 147 (56.5%) and 113 (43.5%) patients, respectively. The proportion of endovascular treatment increased while the open vascular surgery decreased during the study period (*p* = 0.031). In the propensity score adjusted analysis, the performance of CTA was associated with decreased risk of combined major amputation /mortality (odds ratio 0.52, 95% confidence interval 0.27–0.99; *p* = *0.046*) at 1 year.

**Conclusion:** Performance of CTA was associated with a higher amputation-free survival in revascularized patients with Rutherford IIb ALI. CTA seem to provide guidance in selecting the most appropriate candidates for revascularization and choice of technique.

## Introduction

Although important technical advancements have been made, acute lower limb ischemia (ALI) remains associated with high rates of amputation, mortality (1), and reperfusion injuries (2). Emergent revascularization is especially important in Rutherford IIb (3) ALI patients since motor deficit at presentation is associated with poor prognosis. Historically, Rutherford IIb ALI patients have been sent directly to an operative intervention, circumventing any non-invasive imaging, and is still recommended in the medical literature (4). However, a more modern approach has begun to take form resulting in more clinicians choosing to manage patients with Rutherford IIb ALI, like Rutherford I and IIa ALI, by performing imaging, often computed tomography angiography (CTA), prior to intervention.

Intra-arterial thrombolysis (IAT) was already in the 1990's shown to be equally effective as open surgery for the treatment of ALI (5). Techniques and equipment used in endovascular surgery have evolved rapidly since the 1990's which has resulted in a shift toward a broader use of this less invasive method (6). There are, however, still concerns about using IAT in Rutherford IIb ALI since revascularization is more gradual and takes a longer time compared to open revascularization techniques. Nevertheless, patients with Rutherford IIb ALI do undergo IAT (7), but the efficacy and safety compared to open surgery have not been possible to evaluate. In fact, only 30% of 106 included studies in a recent systematic review on ALI reported the clinical presentation according to the Rutherford classification (8).

The main objective of this study was to evaluate if immediate revascularization without performing CTA prior to revascularization in Rutherford IIb ALI is associated with better outcomes.

## Materials and Methods

### Study Population

This study was approved by the Swedish Ethical Review Authority (Diary nr 2020/00764) and informed consent from patients was waived. The study protocol conformed to the ethical guidelines of the 1975 Declaration of Helsinki. Integrity and privacy of each participant were protected and all data is confidential. Patients undergoing open and endovascular revascularization procedures for ALI in the present tertiary referral hospital between January 1, 2006 and December 31, 2018 were included. The study complies with the Strengthening the Reporting of Observational studies in Epidemiology (STROBE) statement for cohort studies (9). Among 681 patients, 260 (38.2%) with Rutherford IIb (motor deficit in the lower limb) ALI at admission were included in the present study.

### CT Angiography

In emergencies, multi-detector row CT was performed from 2004 and onward (10). Run-off CTA scanning was done from hemidiaphragms to the forefoot. Iohexol 90 ml 350 mg I/ml (Omnipaque 350 mg I/ml, GE Healthcare Limited Little Chalfont, England) followed by 50 ml saline flush at flow rate 5 ml/s were injected *via* an 18 G intravenous cannula placed in an antecubital vein. Arterial phase images were obtained 5 s after bolus detection in the suprarenal aorta (threshold 120 HU for Siemens Somatom Definition Flash and threshold 180 HU for Canon Aquilion One). The scanning was done in two series: first from the level of the right atrium to the middle of the femur and secondly from hips to forefoot. The images were reconstructed with 1 and 3 mm thickness in an axial plane. A reconstruction in the coronal and axial plane with 2 mm thickness was also done. Images of the abdomen were reconstructed with 3 mm thickness in the sagittal plane.

### Variable Definitions

Symptom duration was defined as the number of hours from symptom onset until the start of the revascularization procedure. Embolic events resulting in ALI was mainly based upon CTA or angiographic appearance of embolic clots, synchronous embolism to other arterial territories, previous arterial embolism, atrial fibrillation, or other source of embolism. Acute on chronic limb ischemia was defined as an acute exacerbation of existing chronic limb ischemia (claudication, rest pain, or foot ulcer). Major amputation was defined as amputation above the foot level. Only major amputation of the limb affected by ALI was taken into consideration. Major bleeding was defined as hemorrhage resulting in the requirement of blood transfusion, surgery, resulting in stroke or cessation of thrombolysis due to bleeding (11). Anemia was defined as hemoglobin <134 g/L in men and <117 g/L in women, and renal insufficiency was present if serum creatinine reached levels >105 μmol/L in men and >90 μmol/L in women.

### Follow-Up

In five patients, no follow-up data regarding limb status at 1 year was possible to retrieve. Two patients living in a foreign country were not possible to follow up at all. Survival status for 258 (99.2%) patients was searched through the national population registry.

### Statistical Methods

Statistical analysis was performed using IBM SPSS Statistics for Macintosh, version 26.0 (IBM Corp., Armonk, N.Y., USA). Group comparison of nominal data was performed using Pearson's chi-square. Annual time trends for non-invasive imaging prior to revascularization, mode of revascularization (endovascular first therapy or open vascular surgery first), or combined major amputation/mortality at 1 year were assessed using the Kendall's tau-b test. Continuous data were expressed as median and interquartile range (IQR) and comparison between groups was analyzed using the Mann-Whitney U-test. *p* < 0.05 was considered statistically significant.

### Propensity Score Adjusted Analysis

A propensity score technique to adjust for multiple risk factors (12, 13) was used since multivariate adjustments by logistic regression are limited by the number of endpoints, and a limited number of covariates should be modeled (14). With this method, several risk factors for the adverse outcome are used to calculate a propensity score, reflecting the differences in risk factors between those examined with CTA or not. In the next step, the propensity score can be used to adjust for differences between those examined with CTA or not in an analysis of outcomes. In the first step, we used a logistic regression model, with age, sex, hypertension, diabetes mellitus, ischemic heart disease, cerebrovascular disease, atrial fibrillation, claudication, renal insufficiency, anemia, existing foot ulcer, treatment with acetyl salicylic acid (ASA), supra or infra-inguinal arterial occlusion, native artery occlusion, bypass graft occlusion, endoprosthesis occlusion, embolus, open or endovascular surgery, former (2006–2012) or latter (2013–2018) study time period as independent variables, and examination with CTA or not as the dependent variable. Propensity score distribution was trimmed and individuals within the 5–95th percentile were included in the analysis to exclude outliers. Missing data were coded as separate dummy variables and included in the propensity score adjusted analysis. The propensity score variable was inserted as another covariate together with examination with CTA or not in the final logistic regression analysis of outcomes.

## Results

### Annual Time Trends in Rutherford IIb ALI

**Figure 1:**
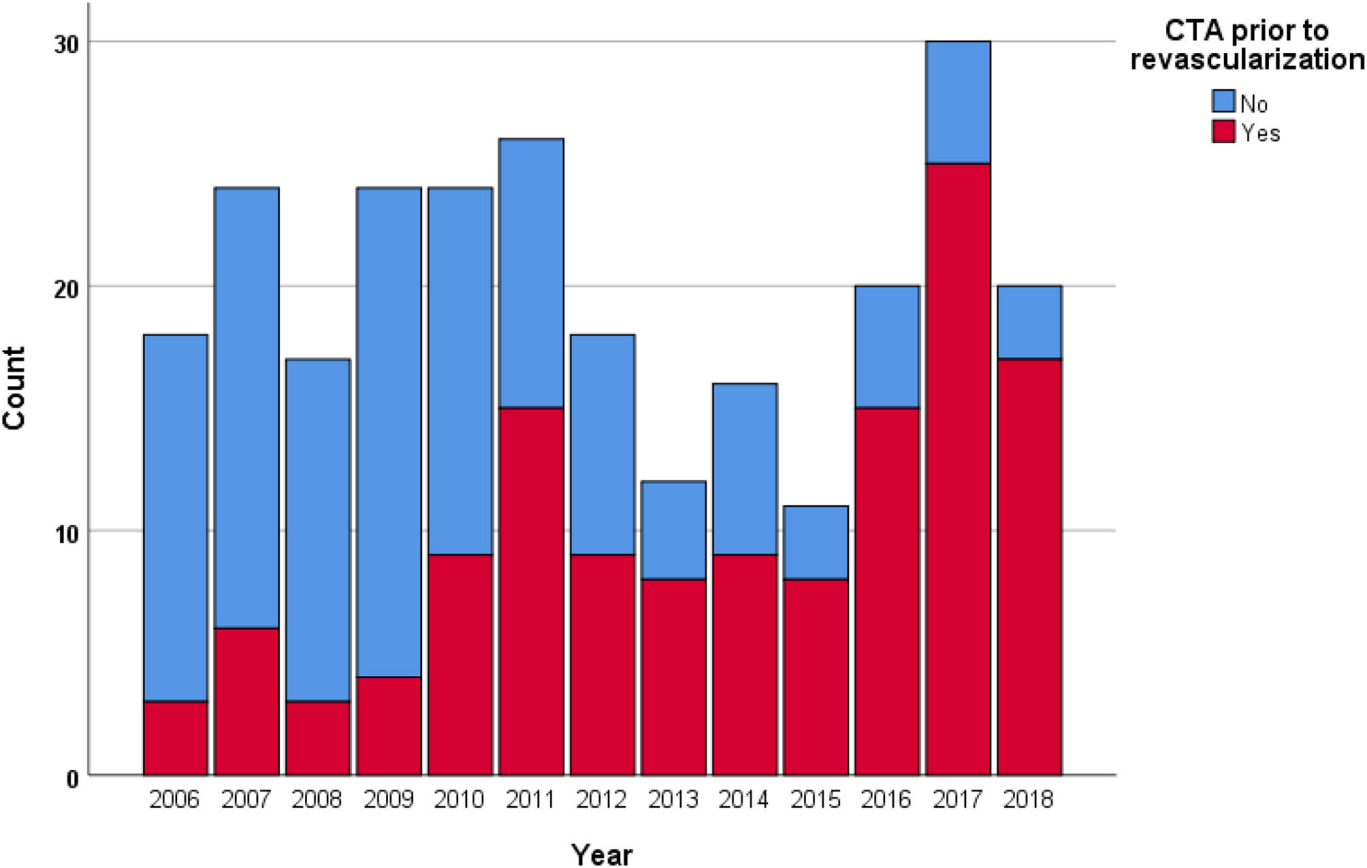
The proportion of computed tomography angiography (CTA) prior to revascularization in Rutherford IIb ALI increased (*p* < 0.001) throughout the study period.

The proportion of non-invasive imaging prior to revascularization (*p* < 0.001), CTA prior to revascularization (*p* < 0.001; Figure 1), endovascular treatment (*p* = 0.031), and amputation-free survival rate at 1 year (*p* = 0.002) increased in Rutherford IIb ALI between 2006 and 2018.

### Non-invasive Imaging Prior to Revascularization in Rutherford IIb ALI

No imaging and any imaging prior to revascularization were performed in 28% (74/260) and 72% (186/260), respectively. The frequency of duplex, magnetic resonance (MR) angiography, and CTA were 16.2% (*n* = 42), 8.5% (*n* = 22), and 50.4% (*n* = 131), respectively. The use of CTA increased throughout the study period (*p* < 0.001), while duplex decreased (*p* = 0.029) and MR angiography was unchanged (*p* = 0.956). CTA prior to revascularization was done more often (*p* = 0.023) among those undergoing endovascular (58.4%; 66/113) compared to open surgical treatment (44.2%; 65/147). This significant difference was not maintained in the latter half (2013–2018) of the study period, 76.8% (43/56) vs. 73.6% (39/53), respectively (*p* = 0.699).

### Mode of Revascularization in Rutherford IIb ALI

Open vascular and endovascular surgery was first performed in 147 (56.5%) and 113 (43.5%) patients, respectively. The primary open vascular procedures were thromboembolectomy (*n* = 140) and bypass [Axillo-bifemoral bypass (2), femoro-popliteal bypass below knee (2), femoro-distal bypass (3); *n* = 7]. The primary endovascular procedures were thrombolysis (*n* = 102), mechanical thromboembolectomy with the Angiojet® device (MEDRAD, Warrendale, Pennsylvania, USA; *n* = 5), primarily stent grafting without thrombolysis (*n* = 4), endovascular aspiration embolectomy (*n* = 1), and primary hybrid revascularization (thrombendarterectomy of the common femoral artery and stenting of the ipsilateral common iliac artery; *n* = 1).

### Characteristics in Patients Examined With CTA or Not Prior to Revascularization

Factors associated with no examination with CTA prior to revascularization with Rutherford IIb ALI were higher age (*p* = 0.011), female gender (*p* = 0.035), atrial fibrillation (*p* = 0.011), infra-inguinal as opposed to supra-inguinal occlusion (*p* < 0.001), native artery occlusion (*p* = 0.014), embolus (*p* = 0.033), open vascular as opposed to endovascular surgery (*p* = 0.023), and former as opposed to latter study period (*p* < 0.001) (Table 1). Among 37 female elderly (≥80 years) patients with atrial fibrillation and infra-inguinal occlusion, 11 were examined with CTA and 26 were not, and the corresponding combined major amputation/mortality at 1 year was 18.2% (2/11) vs. 50.0% (13/26), respectively (*p* = 0.072).

**Table 1:** Characteristics in patients undergoing computed tomography angiography (CTA) or not prior to revascularization in patients with Rutherford IIb acute lower limb ischemia (ALI).

	**CTA**	**No CTA**	
**Variables**	***n =* 131**	***n =* 129**	***p*-value**
Median age in years (IQR)	74 (67–81)	78 (70–86)	0.011
Male gender (%)	73 (55.7)	55 (42.6)	0.035
Hypertension (%)	101 (77.1)	105 (81.4)	0.39
Diabetes mellitus (%)	28 (21.4)	31 (24.0)	0.61
Ischemic heart disease (%)	41 (31.3)	46 (35.7)	0.46
Cerebrovascular disease (%)	25 (19.1)	27 (20.9)	0.71
Atrial Fibrillation (%)	37 (28.2)	56 (43.4)	0.011
Previous claudication (%)	46 (35.1)	46 (35.7)	0.93
Renal insufficiency (%)	47/130 (36.2)	46/128 (35.9)	0.97
Anemia (%)	25/130 (19.2)	32/127 (25.2)	0.25
Existing foot ulcer (%)	8 (6.1)	4 (3.1)	0.25
Medication with acetyl salicylic acid (%)	66 (50.4)	52 (40.3)	0.10
Median symptom duration in hours (onset to start of revascularization) (IQR)	24 (9–72; *n =* 129)	24 (8–72; *n =* 128)	0.70
Acute on chronic limb ischaemia (%)	16 (12.2)	14 (10.9)	0.73
Supra-inguinal arterial occlusion	57 (43.5)	29 (22.5)	
Infra-inguinal arterial occlusion	74 (56.5)	100 (77.5)	<0.001
Native artery occlusion (%)	88 (67.2)	104 (80.6)	0.014
Bypass graft occlusion (%)	14 (10.7)	13 (10.1)	0.87
Endoprosthesis occlusion (%)	32 (24.4)	12 (9.3)	0.001
Embolus (%)	44 (33.6)	60 (46.5)	0.033
Popliteal artery aneurysm (%)	9 (6.9)	17 (13.2)	0.090
Open vascular surgery (%)	65 (49.6)	82 (63.6)	
Endovascular surgery (%)	66 (50.4)	47 (36.4)	0.023
Former study period (2006–2012) (%)	49 (37.4)	102 (79.1)	
Latter study period (2013–2018) (%)	82 (62.6)	27 (20.9)	<0.001

*IQR, interquartile range; CTA, computed tomography angiography; ALI, acute lower limb ischaemia*.

### Outcomes at 1 Year

The overall major amputation rate, mortality, and combined major amputation/mortality at 1 year was 14.9% (38/255), 28.7% (74/258), and 38.7% (99/256), respectively. The major amputation rate, mortality, and combined major amputation at 1 year for those examined with CTA prior to revascularization were 10.2% (13/128), 18.5% (24/130), and 25.8% (33/128), respectively, compared with 19.7% (25/127; *p* = 0.033), 39.1% (50/128; *p* < 0.001), and 51.6% (66/128; *p* < 0.001), respectively, for those not examined with CTA prior to revascularization.

### Propensity Score Adjusted Analysis of Outcomes in Patients Examined With CTA or Not Prior to Revascularization

There was a trend that performance of CTA was associated with a decreased risk of major amputation (OR 0.44, 95% CI 0.18–1.08; *p* = 0.74) at 1 year. Performance of CTA was associated with decreased risk of combined major amputation/mortality (OR 0.52, 95% CI 0.27–0.99; *p* = 0.046) at 1 year (Table 2).

**Table 2:** Propensity score adjusted analysis of outcomes in patients with Rutherford IIb ALI examined with CTA prior to revascularization or not.

**Outcomes at 1 year**	**Propensity score adjusted analysis**	
	**OR (95% CI)**	***p*-value**
Major amputation	0.44 (0.18–1.08)	0.074
Mortality	0.57 (0.28–1.16)	0.121
Major amputation/mortality	0.52 (0.27–0.99)	0.046

*CTA, computed tomography angiography; ALI, acute lower limb ischaemia*.

## Discussion

Performance of CTA was associated with a higher amputation-free survival at 1 year in patients with Rutherford IIb ALI. CTA seem to provide guidance in selecting the most appropriate candidates for revascularization and choice of technique. The favorable results associated with increased use of CTA prior to revascularization in Rutherford IIb ALI justify imaging of the arterial tree in Rutherford IIa (sensory deficit) or I ALI prior to invasive therapy.

The increased proportion of endovascular therapy throughout the study period in Rutherford IIb ALI is not surprising due to the endovascular profile of the present study center. The increasing use of stent and stent graft implantations demands more CTA for detection of endoprosthesis restenosis or occlusions (15) and for subsequent treatment planning. Vascular surgeons have adapted to the fact that CTA has become an integral part of their diagnostic arsenal. Parallel to this, the availability of fast high-quality CT scanners around the clock has increased greatly during the latter part of the study period, making CTA a very useful tool for precise mapping of the extent of the occlusions and stenoses in the lower limb arteries in ALI (16). Hence, these factors have very likely contributed to that more patients with Rutherford IIb ALI were managed by CTA prior to revascularization during the latter half of the study period.

The selection for any treatment might have progressed toward a more restrictive manner regarding revascularization attempts during the latter study period. The significant increase in preoperative CTA during the latter period probably contributed to a higher number of patients with ALI being increasingly refrained from revascularization. It is not uncommon that CTA identifies patients with complex arterial occlusive lesions, multiple arterial emboli to the viscera and limbs, and/or poor run-off in CTA as well as important extravascular findings which make them unsuitable as surgical candidates for revascularization. In a recent report, 38 out of 141 patients with ALI had extravascular findings on CTA of immediate clinical relevance (17). For instance, four patients had previously unknown advanced cancer disease, which is a contraindication for thrombolysis. It is highly warranted to estimate the proportion of patients refrained from revascularization attempt based on CTA findings and/or due to poor performance status prior to the onset of ALI.

The study results showed, typically, that elderly female patients with atrial fibrillation and infra-inguinal occlusion more likely were managed by open vascular surgery in the former study period without having an examination with CTA prior to revascularization. It was not possible to show that this subgroup of patients had a statistically higher combined major amputation/mortality at 1 year, probably due to a statistical type 2 error. The extensiveness and profoundness of motor deficit in the patients with Rutherford IIb ALI were not studied, but it is likely that those with extensive motor deficit were more urgently managed and perhaps more often underwent open vascular surgery without being examined with a CTA prior to revascularization. Interestingly, there was no difference in utilization of emergency CTA at the workup stage between those undergoing open vascular surgery and thrombolysis in the latter half of the study period.

There are limitations to consider when interpreting the results of this retrospective observational study. Even though this study only included patients with Rutherford IIb ALI, it should be acknowledged that the severity and extent of paralysis may vary, which induces treatment selection bias. Similarly, the unknown status of medical therapy and smoking during follow up has introduced residual confounding (18, 19). The proportion of patients with Rutherford IIb ALI turned down for revascularization after CTA during the study period was unknown. The present study was conducted in an endovascular-oriented center with high availability to CT scanners around the clock, which may affect the generalizability of study results.

## Conclusions

Performance of CTA was associated with a higher amputation-free survival in revascularized patients with Rutherford IIb ALI. CTA seem to provide guidance in selecting the most appropriate candidates for revascularization and choice of technique. The results of the present study could be considered when forming or updating future guidelines on the diagnosis of patients with ALI, recommending immediate CTA in patients with Rutherford IIb ALI.

## Data Availability Statement

The raw data supporting the conclusions of this article will be made available by the authors, without undue reservation.

## Ethics Statement

The studies involving human participants were reviewed and approved by the Swedish Ethical Review Authority. Written informed consent for participation was not required for this study in accordance with the national legislation and the institutional requirements.

## Author Contributions

ES was involved in the design of the study, data gathering, data analysis, and writing of the manuscript. RS-B and SA was involved in the design of the study, data gathering, data analysis, and critical review of the manuscript. All authors contributed to the article and approved the submitted version.

## Conflict of Interest

The authors declare that the research was conducted in the absence of any commercial or financial relationships that could be construed as a potential conflict of interest.

## Publisher's Note

All claims expressed in this article are solely those of the authors and do not necessarily represent those of their affiliated organizations, or those of the publisher, the editors and the reviewers. Any product that may be evaluated in this article, or claim that may be made by its manufacturer, is not guaranteed or endorsed by the publisher.
